# A Review on Metallurgical Issues in the Production and Welding Processes of Clad Steels

**DOI:** 10.3390/ma17174420

**Published:** 2024-09-08

**Authors:** Fabio Giudice, Severino Missori, Cristina Scolaro, Andrea Sili

**Affiliations:** 1 Department of Civil Engineering and Architecture, University of Catania, 95123 Catania, Italy; 2 Department of Industrial Engineering, University of Rome-Tor Vergata, 00133 Roma, Italy; missori@uniroma2.it; 3 Department of Engineering, University of Messina, 98166 Messina, Italy; cscolaro@unime.it

**Keywords:** clad steel, filler metal, arc welding, laser beam, welding passes, weld zone, solidification mode, heat-affected zone

## Abstract

Carbon and low-alloy steel plates clad with stainless steel or other metals are a good choice to meet the demand for cost-effective materials to be used in many corrosive environments. Numerous technical solutions are developed for the production of clad steel plates, as well as for their joining by fusion welding. For thick plates, a careful strategy is required in carrying out the multiple passes and in choosing the most suitable filler metals, having to take into account the composition of the base metal and the cladding layer. The specificity of the different processes and materials involved requires an adequate approach in the study of the metallurgical characteristics of clad steel, thus arousing the interest of researchers. Focusing mainly on ferritic steel plates clad with austenitic steel, this article aims to review the scientific literature of recent years which deals with both the production and the fusion welding processes. The metallurgical issues concerning the interfaces and the effects of microstructural characteristics on mechanical behaviour and corrosion resistance will be addressed; in particular, the effects on the fusion and thermally affected zones that form during the fusion welding and weld overlay processes will be analysed and discussed.

## 1. Introduction

Cladding of carbon and low-alloy steel plates with a layer of stainless steel, aluminium or titanium alloy is a cost-effective solution to the growing demand for quality materials coming from various industrial fields, such as the petrochemical, energy production or shipbuilding fields. In this way, the mechanical properties expected from the backing steel can be combined with the corrosion resistance of the cladding metals. In recent decades, to achieve good performance even in aggressive environments, ferritic steels clad with austenitic steels have been successfully utilised in the petrochemical and energy industries for the production of vessels and heat exchangers and for making longitudinally welded clad pipes.

The cladding material can be applied through surface welding (by single- or multi-pass conventional arc welding processes), plate remelting by laser beam welding (LBW), explosion bonding and solid-state welding by coextrusion of cylinders or hot rolling bonding of plates. The last method is suitable for large-scale industrial production (for a review, see the article by Wang et al. [1]). In particular, the hot rolling process is widely utilised to produce cladding layers of austenitic steel on carbon steel plates, which is a cheap solution to meet the requirements of corrosion resistance typical of the heat exchangers and pressure vessels. These plates combine the good mechanical properties of low-cost carbon steel with the high corrosion resistance and heat resistance typical of stainless steel; furthermore, the strong interface bonding makes clad plates fit for further forming processes by plastic deformation or welding [2].

Even if austenitic stainless steel clad plates are the most commonly used for structures in corrosive environments, cladding metals can also be selected from a wide variety of alloys, which provide specific properties expanding the fields of use of the base material. In particular, joining aluminium to steel is of high economic and technical interest for many industries, such as shipbuilding, since it combines the light weight of Al with the low cost and the high structural strength of steel. Transition joints between structural steel and aluminium alloys are currently produced by explosive welding, which is a joining process where the high strains and temperatures, acting in a short period of time, make it particularly suitable for dissimilar materials with very different metallurgical properties [3].

Generally, clad steels show interfaces with strong metallurgical bonding, even if some critical issues have been highlighted in the literature: for example, regarding stainless steel clad plates produced by hot rolling, the article by Dhib et al. [4] deals with the formation of a hard carburized layer due to the carbon diffusion towards the austenitic stainless steel, in which the precipitation of Cr-carbides occurs, with harmful consequences for corrosion resistance [5].

The fundamental requirement for clad steel weldments is to obtain a continuous layer that maintains the corrosion-resistant characteristics of the cladding material; however, in fusion welding, the significant differences in composition between the deposited layers are a source of drawbacks that could make the use of clad steels challenging. As documented in the literature (see the review by Wang et al. [1] and, more specifically, the articles cited in Section 3), several procedures based on the use of filler wire, applied via conventional arc welding or hybrid laser beam/arc welding, have been successfully developed over the years. However, in some cases they have been affected by issues that are essentially due to dilution phenomena between base steel and cladding alloys, as well as between adjacent layers obtained with sequential passes [6]. In thick plates, arc welding is carried out with multiple passes, which involve dilution phenomena and heating cycles, affecting the weld composition and in general the metallurgical characteristics of the fusion zone (FZ) and heat-affected zone (HAZ). In this regard, the addition of consumable inserts, interposed between the edges of two butt-positioned clad plates, has been proposed as an advantageous solution for LBW in a single pass to obtain narrow welds with deep penetration [7]. The purpose of this article is to outline a comprehensive analysis of the most recent scientific literature on issues relating to clad steel, focusing mainly on ferritic steel plates clad with austenitic steel.

The metallurgical features of both as-produced plates and welds will be specifically highlighted for their relevance in terms of mechanical properties and corrosion resistance. Therefore, the production processes as well as the various fusion welding techniques will be reviewed in Section 2 and Section 3, respectively, to analyse the effects on clad steel properties. The main metallurgical issues in weld overlay and fusion welding will be debated in Section 4, with particular regard to those arising from dilution and solidification modes. Finally, conclusions and future research directions will be outlined in Section 5. 

## 2. Metallurgy of As-Produced Clad Steel Plates 

### 2.1. Hot Rolled Clad Steel Plates

**Figure 1:**
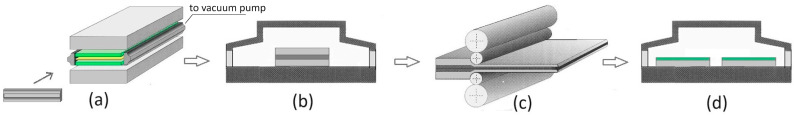
Hot rolling process of two sets of base steel (grey) and cladding layer (green) separated by an interposed layer (yellow): (**a**) assembling of the “sandwich”-type composition; (**b**) preheating; (**c**) hot rolling; (**d**) final annealing of the clad plates.

Hot rolling is widely used for manufacturing clad plates, as it is an economic and efficient process for mass production. The thickness of the cladding layer, in general austenitic stainless steel, can be up to 6–7 mm, while that of the base carbon or low-alloyed steel is usually three or four times thicker [1]. With reference to Figure 1, rolling is performed on a pack of plates arranged in a sandwich-type composition: two sets of cladding layers/base steel are symmetrically assembled with an intermediate separating layer, then four bars are welded all around their edges to form a sealed chamber in which a vacuum can be obtained through an exhaust hole connected to a vacuum pump [8]. In this way, the formation of oxides, with consequent deterioration of the interface bonding, is avoided. An interlayer between the base steel and cladding is also added to prevent the formation of intermetallic compounds, thereby improving the bonding strength of the composite plate [9]. 

During the bonding process, the plates undergo some thermal cycles, which in the case of ASTM A283 grade C carbon steel (base metal) and AISI 316 austenitic steel (clad layer) consists of the following steps [10]:

- Preheating of the assembled pack, with a slow increase from room temperature up to 1230 °C, maintained for 5 h;
- Hot rolling between 1230 and 850 °C, followed by air cooling;
- Disassembling of the rolled pack;
- Final annealing in the range 920–950 °C, followed by air cooling.

Hot rolling gives rise to a continuous bound between the two metals, which maintain their composition unchanged, except in a narrow band at the interface where the permanence at high temperature activates diffusion. A thin and slight wave-like interface, the so-called cladding line, separates the ferritic/pearlitic microstructure of the base carbon steel from the typical austenitic microstructure of the cladding layer. The non-perfect linearity of the interface is a typical phenomenon of instability, due to the high rolling temperature and reduction ratio [2].

An ideal cladding interface should be integral and continuous without any defects, such as inclusions or unbonded areas which could affect the mechanical properties, as shown by Li et al. [11], who studied the interfacial fracture evolution in austenitic stainless steel clad plates.

The interfacial bonding strength is affected by several factors, such as the bonding temperature, the deformation reduction ratio, the roll speed and the interfacial oxidization. In this regard, Zhu et al. [12] found that the interface bonding strength and interface toughness can be affected by the presence of oxides positioned along the cladding line, which may act as alternative crack propagation paths in shear tests. Complete metallurgical bonding can be obtained at high temperatures and in a protective atmosphere or in a vacuum. 

Compared with cold roll bonding, hot rolling allows easy bonding of the two metals with a slight deformation in thickness, due to the effects of plastic deformation and recrystallization. However, the bond strength decreases drastically when the metal surfaces are oxidized; therefore, their preparation by appropriate mechanical polishing and chemical etching [10] is a key factor for the optimal success of the bonding process [13].

**Figure 2:**
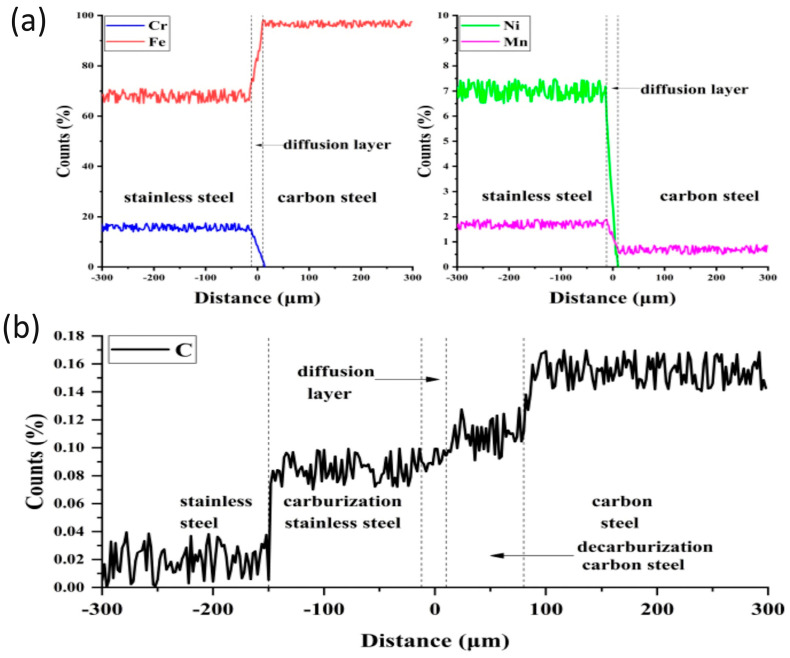
Results of electron probe micro-analyser measurements across the interface SUS304/Q235: (**a**) Cr, Fe, Ni and Mn; (**b**) carbon. Reproduced from [5].

The permanence at a high temperature of the plates generates diffusion phenomena across the cladding line. The substitutional alloy elements, of which the austenitic layer is rich, diffuse towards the backing carbon steel, while the small interstitial atoms of carbon migrate more quickly in the opposite direction, thus affecting a larger zone [4]. As shown by the diffusion profile in Figure 2, the interface between the two steels is characterised by a diffusion layer, 20 µm thick, where the contents of Cr, Ni and Mn decrease linearly from the stainless steel side to the carbon steel one, whereas the content of Fe has an opposite trend; furthermore, the mobile carbon atoms diffuse over a longer distance towards the austenitic steel [5].

**Figure 3:**
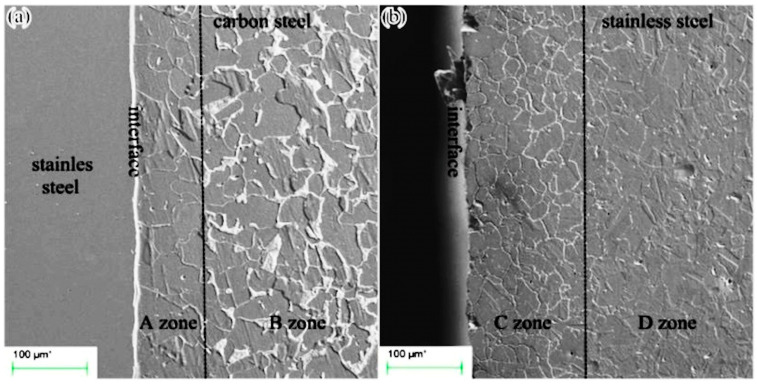
Clad plate interface: (**a**) Q235 carbon steel side; (**b**) SUS304 austenitic steel side. Reproduced from [5].

Due to diffusion in hot rolled clad plates, a decarburized zone consisting of coarse ferritic grains (in which pearlite is absent) and a carburized layer are formed on the carbon steel and austenitic stainless steel sides, respectively. With reference to Figure 3, the following areas can be identified at the carbon steel/austenitic steel interface [5]: a decarburized layer near the interface, mainly composed of ferrite (A zone); carbon steel far from the interface consisting of ferrite and pearlite aligned along the rolling band (B zone); a carburized layer at the austenitic steel side, characterised by Cr-carbide precipitation at the grain boundary (C zone); and an unaffected austenitic steel microstructure (D zone).

**Figure 4:**
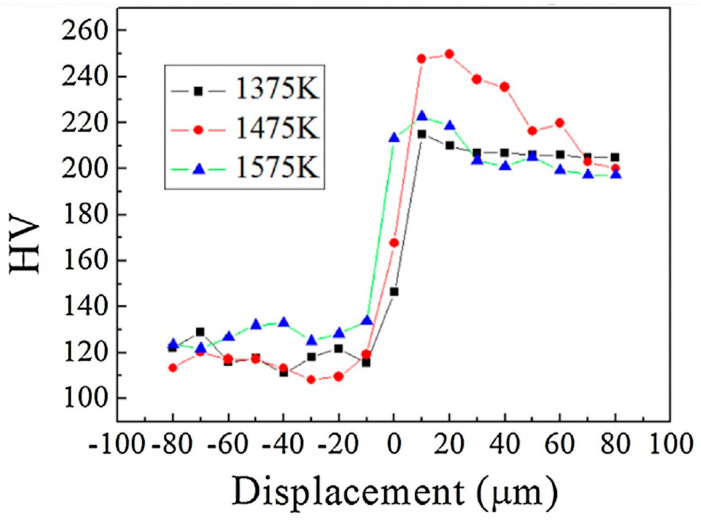
Vickers microhardness profile along a transversal line across the interface, from the Q235 carbon steel side (on the left) to the SUS 304 austenitic steel side (on the right), for three different rolling temperatures. Reproduced from [15] with permission from Elsevier.

The diffusion coefficient of carbon is several orders of magnitude larger than that of chromium; therefore, the carbon atoms readily diffuse through the bonding interface, whereas the diffusion of Cr is much more difficult [14]. Hence, carbon atoms diffuse into the austenitic stainless steel layer, where Cr-carbide formation occurs during cooling in the sensitizing temperature range 550–850 °C, causing a sharp increase in hardness near the cladding line, and maintaining high values within the carburized layer at the austenitic steel side. This is confirmed by the results of the Vickers microhardness surveys performed along a line transverse to the interface, such as in the case shown in Figure 4: the hardness peak increases up to 1475 K, as long as the effect due to carbon diffusion prevails, whereas higher temperatures cause a softening of the matrix and consequently a reduction in hardening, which becomes evident at 1575 K [15]. 

In any case, the tensile properties of the hot rolled plates change significantly as a function of the rolling temperature or time. To overcome this issue, Yang et al. [16] proposed a new method based on a liquid–solid bonding process, in which stainless steel is deposited through a melting crucible onto a carbon steel plate. The clad plate, subsequently hot rolled at 900 °C, results in a superior combination of high shear strength and ductility of the cladding interface.

**Figure 5:**
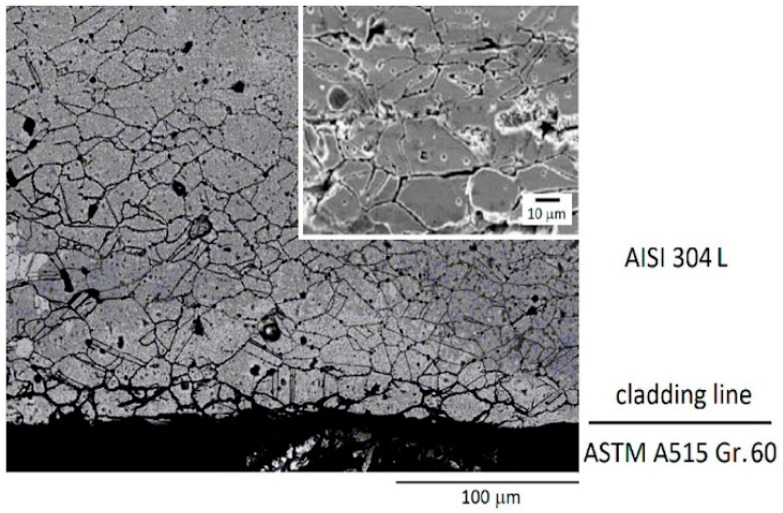
Sensitization to the intergranular corrosion of the carburized zone near the interface of carbon steel/austenitic steel (the higher magnification in the box shows the effect of corrosion immediately close to the cladding line). Reproduced from [18].

Cr-carbide precipitation causes Cr depletion at the grain boundary. Here, the susceptibility to intergranular corrosion can significantly increase, especially when the Cr content is reduced to values lower than 12 wt.% [17]. The effects of sensitization to intergranular corrosion on the austenitic steel side near the cladding line are shown by the micrograph in Figure 5 [18], taken after the ASTM Test A262-Practice E [19] was carried out.

The width of the zones affected by diffusion depends on the processing temperature, as documented by Liu et al. [20] in the case of Q235 carbon steel plates with SUS 304 austenitic steel cladding layers, assembled in a vacuum, maintained for 2–3 h at three different temperatures (1100, 1200 and 1300 °C) and then hot rolled. They observed a reduction in ultimate tensile strength (σ_UTS_) and an increase in elongation (ε) as the preheating temperature increased from 1100 to 1300 °C: in particular, σ_UTS_ varied from 578 ± 8 to 528 ± 8 MPa and ε from 46 ± 3 to 60 ± 3%. This is due to the formation of a strong interface that delays the propagation of delamination cracks and localized necking. 

Considering that the hot rolled plates could suffer deformation-related defects, vacuum diffusion bonding is a good alternative for small productions. This process is performed in a vacuum chamber by applying pressure at a high temperature. Therefore, it allows one to manage the main working parameters, such as temperature, pressure and time, obtaining further improvements in the interfacial bonding strength, as demonstrated by Li et al. [21]. The authors achieved good metallurgically bonded interfaces in stainless steel clad plates; they experimented with the effects of the bonding temperature (ranging from 700 to 1100 °C) and time (from 1 to 4 h), demonstrating that the best mechanical properties were obtained when the manufacturing process was carried out under a pressure of 15 MPa, at 800 °C and for 120 min. 

Concerning post-processing heat treatment, it is noteworthy to consider that stainless steel generally requires temperatures above 1000 °C to dissolve deleterious secondary phases which may form in the range 600–100 °C, while the mechanical properties of carbon steel get worse when treated above 1000 °C because of grain growth. Therefore, it is necessary to determine, for each specific case, the optimal treatment conditions for achieving a balance between the properties of the two metals. For example, Song et al. [22] developed a heat treatment for a clad steel to be utilised for hull structures in shipbuilding (S32750 cladding metal/EH40 base metal EH40), consisting of water quenching after treatment at 1080 °C for 1 h/in and tempering at 550 °C for 1–2 h followed by air cooling.

### 2.2. Explosion-Welded Clad Steel Plates

It is a fact that joining aluminium to steel is challenging for metallurgy, due to the wide difference in their material properties (especially in the melting point) and the high susceptibility to generating brittle intermetallic phases, which make the use of conventional welding techniques very difficult. 

For these reasons, both the volume of the interacting materials and the permanence at a high temperature should be minimised. High-velocity impact processes meet these requirements; moreover, they can be performed without the addition of an external heat input (for a review, see the article by Wang et al. [23]). 

Among these processes, explosive welding, in which a pressure wave pushes with high velocity the so-called flyer plate against the back or base plate, is currently utilised to produce joints between metals that are otherwise non-weldable. The exceptional mechanical properties of the weld depend on the way the two surfaces are brought into contact, as the exchange of valence electrons makes the formation of interatomic bonds possible. In any case, explosive welding is characterised by an extremely short duration, which minimises heat dissipation and prevents the formation of the heat-affected zone (HAZ) typical of fusion welding [24].

**Figure 6:**
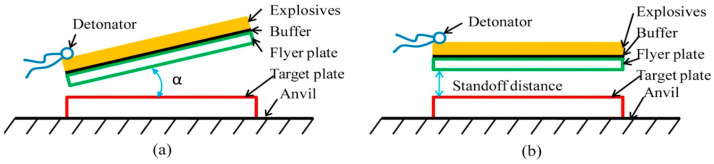
Sketch of the experimental setups for explosive welding: (**a**) inclined; (**b**) parallel. Reproduced from [23].

Explosive welding of plates is generally carried out under inclined or parallel mode, as shown in Figure 6; a buffer was used to prevent the flyer plate from being damaged by the explosion. The inclined setup, under the initial angle α, was introduced first, whereas the parallel one was developed later to weld large plates with a pre-determined standoff distance. The jet sweeps away the oxide films on the surfaces of the two metallic plate metals, favouring the formation of metallurgical bonds on an atomic distance scale [25].

The transition joints between structural steel and Al alloy are useful in shipbuilding and in general when it is necessary to connect structures made of such different metals [26]. For their production, the best results are achieved using an explosive mixture (with low detonation velocity and a low explosive ratio) and an intermediate layer made of commercial pure aluminium [27]. 

**Figure 7:**
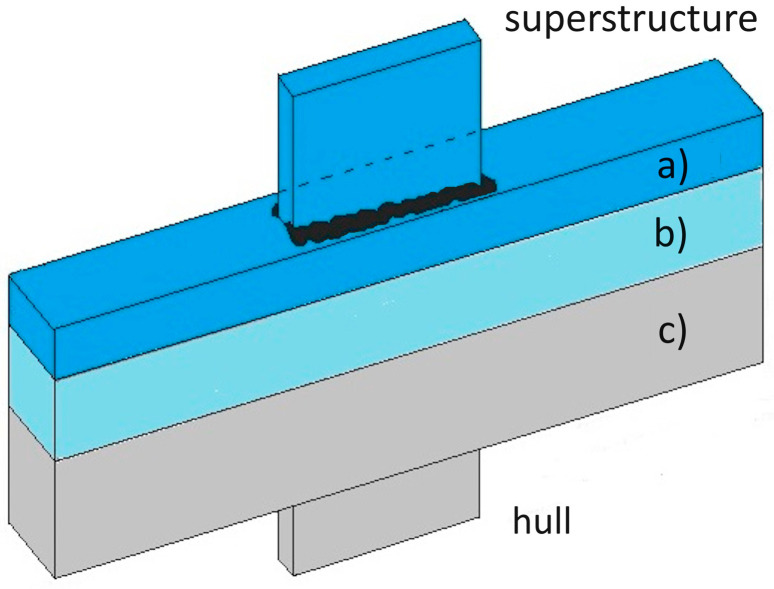
Triclad joint: (**a**) AA5083 Al alloy; (**b**) AA1050 Al interlayer; (**c**) ASTM A516 Gr 55 structural steel.

This hybrid connection is becoming increasingly successful in shipbuilding as it allows the joining in turn, by conventional fusion welding, of the steel substrate to the steel hull and similarly the Al alloy layer to any light alloy superstructures (Figure 7). 

Several articles in the literature deal with the thermomechanical conditions and the process parameters: in the case of the Al-Fe system, Carvalho et al. [28] showed how the detonation velocity (experimentally measured in the range 2000–3000 m/s), with the calculated values of the impact velocity (about 300–400 m/s) and collision angle (from 7 to 10°), influences the final microstructure with the formation of embrittling intermetallic phases.

**Figure 8:**
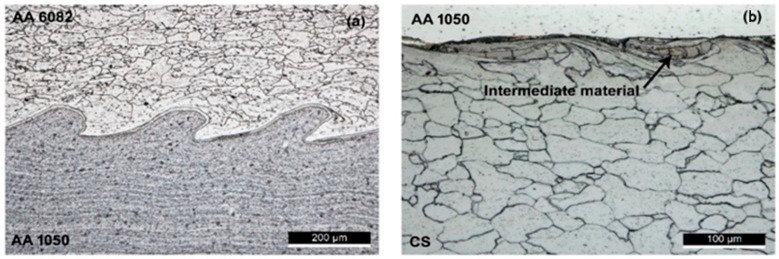
Micrographs of the weld interface: (**a**) AA6082 AA1050 interface; (**b**) AA1050/carbon steel interface. Reproduced from [27].

The morphology of the weld interface has been characterised by many authors. In some cases, the pressure shockwave due to the explosion causes a wavy profile, which has been documented in the literature for different joints, for example Al/Al alloy (Figure 8a) [27], CuCrZr/316L [29], AISI 316/Q235 B [30] and Cu-DHP/AISI 304L [31], whereas, in other cases, such as for an Al/carbon steel joint [27], the interface is almost flat (Figure 8b). For the conditions that lead to the formation of a wavy or straight interface, see also the numerical approach carried out by Ayele et al. [32] for a bimetallic composition (Al/carbon steel), and by Campanella et al. [3] for a joint with three dissimilar materials (AA5083 aluminium alloy and A516 steel, with an intermediate layer made of AA1050 aluminium alloy).

In this regard, Carvalho et al. [33] demonstrated that the AA 1050 interlayer improves the weldability of the AA 6082 flayer plate to the AISI 304 base plate, while it has no beneficial effects on the mechanical properties of the aluminium-to-carbon steel welds. However, the formation of embrittling intermetallic compounds can be observed at the interface between aluminium and steel, as indicated in Figure 7b. Therefore, attention must be paid, as these embrittling phases could grow in the case of subsequent thermal cycles resulting from any welding processes [34]. 

Explosive welding has proven to be suitable for joining aluminium and steel plates on large scales up to metres. It is currently utilised in shipbuilding to produce the Triclad transition joint consisting of ASTM A516 Grade 55 structural steel as the base plate and AA 5086 or AA 5083 aluminium alloy as the flyer plate, with AA 1050, almost pure aluminium, as the interlayer [35]. 

In the Triclad joint, as shown in [36], the interface on the side AA1050/A516 Grade 55 shows a linear profile with some particles recognized as an Al-Fe intermetallic phase, while on the opposite side, it is characterised by a wavy morphology that interlocks the Al/Al alloy interface, resulting in an increase in toughness and strength. 

The wavy morphology depends on the process conditions, as demonstrated by Campanella et al. [3], who developed a numerical method for determining the parameters that affect the peak height and wavelength in the Triclad joint, as well as by Lee et al. [37], who simulated the wave formation in the Cu-Ti system.

### 2.3. Weld Overlaid Clad Steel Plates

For several decades, the weld surface cladding process of steel tubes or plates has been performed mainly by depositing the filler metal in the form of wire, usually molten by conventional arc welding [38]. This process, also called weld overlay, provides a cost-effective application of a layer at least 3 mm thick, in agreement with [39], whose final composition is determined by dilution between the filler and base metal. The overlaid layer is characterised by strong metallurgical bonding with the base material, due to the interpenetration of the deposited and supporting materials, which can be obtained with various traditional processes [40]; however, the formation of partially diluted zones (PDZs) at the interface between the cladding layer and base steel could be a potential issue, which will be discussed in Section 4.

The quality of cladding is affected by the weld bead geometry, which in turn depends on process parameters of arc welding, such as current, voltage and traveling speed, as highlighted by Saha et al. [41]. They performed the deposition of AISI 316 weld beads on a low-alloy structural steel plate using the gas metal arc welding (GMAW) process with 100% CO_2_ as a shielding gas, experimenting with different combinations of current, voltage and arc travel speed, chosen so that the heat input increases from 0.35 to 0.75 kJ/mm. The GMAW process was also utilised by Aslam et al. [42] to deposit an AISI 304 layer on the surface of a mild steel plate. These authors developed a transient thermal numerical model to identify the effect of the welding parameters on the clad bead geometry.

Many authors have carried out experimental works to characterise the cladding obtained by weld overlay (see [43] for an overview of the different processes utilised and the layer characterisation): e.g., Sowrirajan et al. [44] investigated the effects of weld dilution on the thermal conductivity of austenitic stainless steel layers deposited by flux-cored arc welding (FCAW) on structural steel plates; Moreno et al. [45] focused their experimental work on the effects of FCAW parameters to qualify a martensitic weld bead deposited on AISI 1020 base steel; and Cattivelli et al. [46] studied any potential connection between the residual stresses generated during cladding through submerged arc welding (SAW), those generated during the post-weld heat treatment (PWHT) and the propensity for underclad cracking.

In general, the composition of a weld overlaid layer is characterised by a certain amount of dilution, due to the contribution of both the filler and base metal. Recently, Mattias et al. [47] developed a process of gas tungsten arc welding (GMAW) for depositing through three passes, on an ASTM A516 Gr. 70 carbon steel plate, a layer of super austenitic stainless steel containing 6% of Mo, the result of which was able to provide acceptable corrosion performance. The authors obtained a layer with a global dilution of 11% through the application of a low heat input, achieving high cooling rate and helping to minimise the secondary phases in agreement with the results shown in [48] on the solidification and segregation characteristics of a super austenitic stainless steel. The formation of cracks, due to metal shrinkage, could occur during the cooling stage. This drawback can be avoided by adding a buffer layer along with the multilayer deposition or by performing appropriate preheating of the electrode [43]. 

The electroslag welding (ESW) process is proposed as a valuable alternative for overlay welding applications, due to the possibility of obtaining a thinner cladding resulting in high productivity and significant cost reduction. This process was used in [49] to deposit a single layer of AISI 904 L on a substrate of ASTM A516 Gr. 70. The authors demonstrated that, due to its superior deposition rate, the ESW process can provide weld claddings with adequate microstructure and corrosion resistance together with increased productivity and cost reduction compared to the usual arc welding processes.

Recently, the gas metal arc welding process with rotating electrode (GMAW-RE) has shown to be promising, since the rotational movement of the wire-electrode provides more homogeneous weld bead profiles, suitable to weld overlaying, as shown by Costa et al. [50]. In their work, the feasibility of the GMAW-RE in depositing a single layer of Inconel 625 weld cladding with suitable properties was investigated. They obtained a homogeneous weld penetration profile, low dilution ratio and adequate reinforcement, thanks to an austenitic microstructure containing a low number of secondary phases.

The hot wire gas tungsten arc welding (GTAW-HW) process benefits from preheating the filler wire before reaching the melt pool. This process has proven to provide high quality, versatility and low cost with an appropriate setting of process parameters [51]. It was applied by Conzaga et al. [52] to weld overlay an API 5L X65 steel pipe with a 70%Ni30%Cu alloy by HW-GTAW. The microstructural and mechanical characterisation performed by the authors demonstrated promising results for applications in seawater systems.

**Figure 9:**
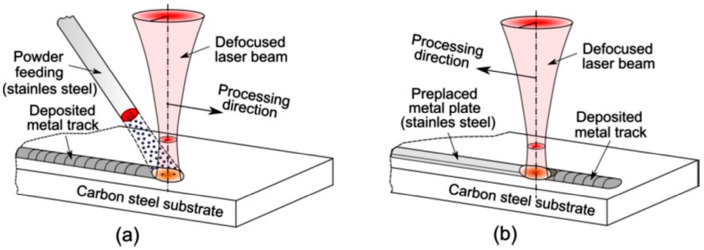
Weld overlay of carbon steel plates: (**a**) conventional processing using wire or powder as feedstock; (**b**) modified processing using a scrap plate as feedstock. Reproduced from [53].

In the usual weld overlay processes, the filler is deposited on the base metal by melting a consumable wire via an electric arc or electro slag process. Nowadays, laser direct metal deposition is proving to be a prominent technique, in which the filler, in the form of a metallic powder, is sprayed over the substrate and sintered (Figure 9a). A laser beam is a flexible tool for melting metals in different forms; therefore, a possible filler for cladding metal could be waste materials in the form of a plate, machined at a given thickness. In this regard, Bunaziv et al. [53] carried out the remelting of 2.0 mm thick 316 L stainless steel plates placed on a carbon steel substrate, using a high-power fibre laser beam (Figure 9b). This process, which allows one to achieve an acceptable corrosion resistance, is inexpensive and promising since scrap metals can be reused as filler material.

**Figure 10:**
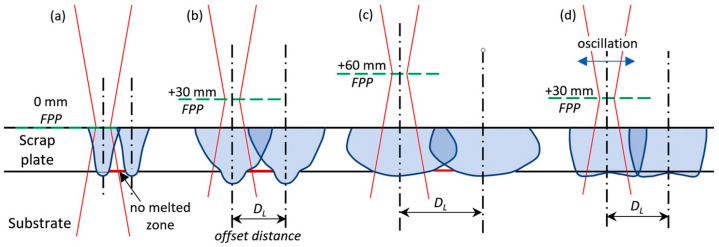
Geometries of two adjacent tracks for different values of FPP: (**a**) narrow track and insufficient overlapping; (**b**) optimization of tracks by defocused laser beam; (**c**) highly defocused laser beam; (**d**) optimal tracks obtained with laser beam oscillations. Reproduced from [53].

As shown in Figure 10, in laser beam overlay, the focal point position (FPP) is a crucial parameter to achieve the optimal track geometry, avoiding the presence of melted zones between two passes and minimising dilution with the carbon steel substrate.

In recent years, due to its excellent stability, precise heat input and high power density, laser beam welding has also proven to be a promising technique for underwater processes, such as welding, weld cladding or laser remelting to repair and improve metal surfaces of marine structures, as shown in the experimental work of Li et al. [54]. 

When working underwater, the irradiated water can strongly shield the laser beam, resulting in a reduction in the absorption efficiency of the laser upon the workpiece surface. Under this condition, the formation of a dry region that acts as a channel around the laser beam is necessary for a successful process. Therefore, a drainage device is needed to produce a dry area on the surface of the damaged marine structure to keep water away and ensure operational flexibility and high manufacturing quality. Wang et al. [55] designed an in-house gas curtain nozzle, which was coupled with the laser cladding head as one underwater production tool. It was utilised to repair a pre-damaged surface of a mill-annealed HSLA-100 steel plate by weld overlaying the filler material consisting of HSLA-100 powder in the form of gas-atomized spherical particles. Other different technical solutions have been developed in the literature, such as drainage methods based on a curtain of gas or water convoyed onto the workpiece surface (for a review on this topic, see [56]). 

## 3. Fusion Welding of Clad Steel

### 3.1. Arc Welding Processes (Hybrid Multi-Passes)

Clad steel plates are usually joined by fusion welding, because other types of fasteners would require unacceptable machining, such as drilling holes for bolting, which could leave the base metal exposed to environmental etching. Currently, various welding methods, such as GTAW, GMAW, SAW or shielded metal arc welding (SMAW), are commonly carried out by depositing multiple layers using traditional multi-pass procedures, as reported below.

In any case, fusion welding of stainless steel clad plates is challenging due to the great diversity in chemical compositions, microstructures and mechanical and physical properties between the substrate and the cladding metal. For this reason, clad plates are rarely welded with a single procedure, even if with different filler metals, such as in [10] where SMAW was used for both the base metal and cladding layer. 

In multi-pass processes, specific working conditions should be applied at the base steel and cladding alloy level. In most cases, welding is performed by different methods, so as to use the one that is most appropriate for the substrate and cladding layer, respectively [57,58]. 

As a matter of fact, there are several issues due to the presence of two metals, each one with its peculiar characteristics, and specific requirements must be considered in the choice of the process setup. Firstly, the effects due to the different values of the linear expansion coefficient (about 12 × 10^−6^ and 17 × 10^−6^ °C^−1^ for carbon steel and austenitic steel, respectively) and thermal conductivity (about 45 and 16 W/(°C∙m) for carbon steel and austenitic steel, respectively) could lead to the onset of residual stress and distortion, as documented in [59]. 

Furthermore, inhomogeneity in the weld composition could occur since the deposited layers undergo dilution with each other, as well as with the cladding alloy and base steel; in addition to this, the formation of diffusion zones of carbon and other alloy elements leads to harmful consequences for the mechanical and corrosion behaviour of the weld. To address these issues, various welding methods have been proposed; they are characterised by the combination of two or more welding processes and filler materials depending on the involved metals, as documented in some significant articles from recent years, cited below. These are essentially composite procedures, defined with the term “hybrid”, being based on multiple passes with different arc welding processes and consequently with the deposition of multiple layers. In general, the welding sequence starting from the base steel provides better joint performance [6].

**Figure 11:**
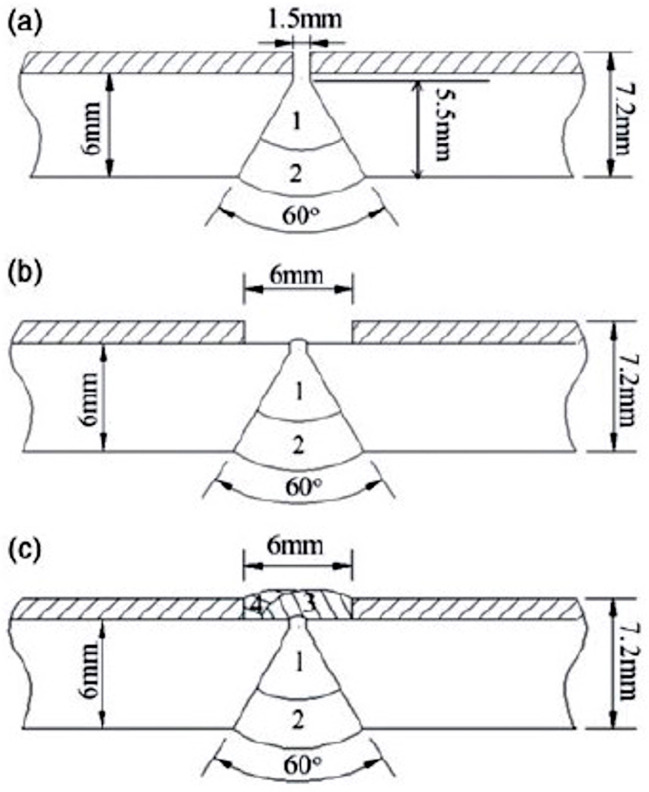
Welding sequences for two butt-positioned plates (Q235 base steel and AISI 304 cladding layer): (**a**) carbon steel backing welding and carbon steel covering welding; (**b**) cleaning the stainless steel cladding; (**c**) stainless steel covering welding. Reproduced from [60] with permission from Taylor & Francis Ltd.

Dhib et al. [10] welded together two A283/A316 clad plates by a multi-pass and multilayer SMAW process, using three filler metals: A283 for the layers close to the A283 base steel, 316 for the layers close to the 316 cladding steel and 309L for the intermediate layers. They compared three different sequences, each one with a number of passes ranging from 7 to 15, obtaining the best results in terms of toughness when performing the 1st passes to seal the base steel. An et al. [60] showed that stainless steel clad plates can be successfully welded using GMAW (with ER70S-6 wire) and GTAW (with ER309L wire) for the Q235 base steel and the AISI 304 clad steel, respectively. They adopted a hybrid procedure in three passes: first, carbon steel backing welding, followed by carbon steel covering welding and, finally, stainless steel covering welding (Figure 11).

In [61], a procedure for welding two plates of Q235 carbon steel clad with AISI 304 austenitic steel (total thickness 7.2 mm) was used experimentally, depositing a transition layer of a suitable filler material to limit Cr and Ni diffusion and the consequent formation of a brittle martensitic zone. First, two passes of GMAW (ER50-6 filler) were carried out on the carbon steel side, then a transition layer was deposited using GTAW with E309L as a filler, or SMAW with the same filler. Finally, the last pass of SMAW (E308 filler) was performed to complete the clad steel side.

In [62], two passes of GTAW were performed on the AISI 321 cladding layer, first with E347 filler for the root pass and then with ER309 filler for the transition layer. Finally, the Q345R carbon steel side was completed using four passes of the SMAW process with E309-16 filler, or otherwise with E4315 to save costs.

In the works by Ghorbel et al. [57,58], three passes of SMAW (E7018 filler) were performed on the A283 Gr C base low-carbon steel; then, the weld was completed by means of the GTAW process with two passes on the transition zone (ER309L filler) and three passes on the A240 TP 316L cladding layer. 

Picchi et al. [63] utilised 3 different welding processes to join two butt-positioned plates of ASTM A516 GR.70 steel clad with AISI 904L austenitic steel (overall thickness 31 mm): Near the cladding layer, 1 pass of GTAW (ER70S-6 filler) as root welding was used; on the carbon steel side, 4 passes of GMAW (ER70S-6 filler) and 15 passes of SAW (F7A2-EM12K filler) were used. Finally, on the AISI 904L side, the joint was completed with electroslag strip cladding using a single pass of Inconel 625 alloy.

Recently, Ban et al. [64] carried out a comprehensive study aimed at metallurgically and mechanically characterising the welds between two bimetallic plates consisting of Q355 structural steel (10 mm thick substrate) and AISI 316 stainless steel (2 mm thick cladding). All butt-welding trials were performed by GTAW with pure Ar as the shielding gas. The use of ER316L filler only was compared with that of different fillers, such as ER50-6 for the structural steel and ER316L for the austenitic steel, which was also experimented with to fill a transition layer. The results of the mechanical tests showed that the use of different fillers allows one to meet the specification requirements and achieve excellent mechanical properties.

### 3.2. Laser and Hybrid Laser/Arc Processes

The traditional arc welding procedures require that base and clad steels are welded separately with different filler materials, to avoid undesirable dilution. Since multiple passes are required to weld clad steels, the overall efficiency of the process is limited and therefore it was deemed worth investigating the possibility of reducing the number of passes using high-penetration LBW. 

In [7], the authors butt-welded two 2205/X65 clade plates, 4 mm thick, using a fibre laser apparatus in a single pass. They obtained a full-penetration weld without any defects; however, the FZ showed a non-uniform composition and a martensitic microstructure with high hardness at the level of the carbon steel. To overcome this shortcoming, in [65] two plates of ASTM A515 grade 60 structural steel clad with AISI 304 austenitic stainless steel (total thickness 9 mm) were butt-welded in an experiment with the use of both a laser beam apparatus and a hybrid laser beam–electric arc setup. Specifically, the laser beam preceded the arc; consequently, two distinct impingement points (with a distance between each other of 55 mm) and two melting pools were generated. 

**Figure 12:**
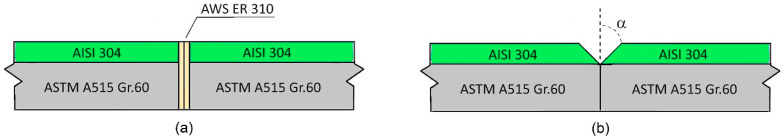
Preparation of the butt-positioned plates: (**a**) interposed consumable inserts for LBW; (**b**) chamfered V-groove for the hybrid LBW–GMAW combination [65].

In the case of the laser beam alone, the filler metal was added as consumable inserts of AWS ER310 in the form of two strips (each one 0.5 mm thick), which were interposed between the butt-positioned plates (Figure 12a), whereas an ER 308 filler wire was used for the hybrid LBW-GMAW combination. In this case, on the AISI 304 side, the plates were bevelled with a V-groove, edges inclined at α = 45° and no gap, as shown in Figure 12b. In both cases, the plates were welded with the clad steel side exposed at the thermal source. In the LBW process, uniform composition and microhardness values were obtained along the longitudinal axis of the FZ. The hybrid process had advantageous results, achieving both deep penetration (due to the laser beam) and better tolerance for geometric defects (due to GMAW).

**Figure 13:**
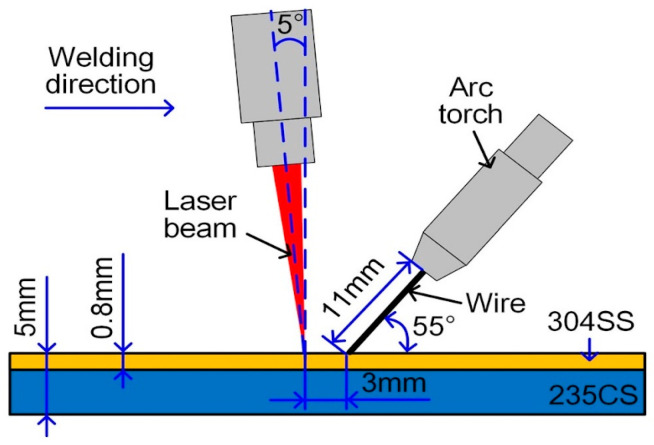
Setup of the hybrid LBW–GMAW process. Reproduced from [66] with permission from Springer Nature.

In [66], a single-pass laser/arc hybrid welding of two AISI 304 stainless steel/Q235B carbon steel plates (total thickness 9 mm) was carried out using ER310 austenitic stainless steel as filler wire. The laser beam and torch were both placed on the clad steel side (Figure 13); in this case as well, the plates were butt-positioned; however, unlike in the previous case, they were prepared with squared edges, and the torch preceded, at a distance of 3 mm, the laser beam. In this work, the authors verified that the laser power value had greater impact on the weld corrosion resistance than that of the wire filling rate.

More recently, some authors proved that the hybrid laser–electric arc process allows welding of thick steel plates (for example, 20 [67] or 25 mm [68]). This method combines the advantages of the large penetration capacity of LBW and the strong adaptability of arc welding to any misalignments of the plates [69], with the results also being useful in enhancing the beneficial effects of laser oscillations on mechanical properties [70].

The considerations on the different welding processes carried out in this section are summarized in qualitative form in Table 1.

## 4. Discussion

The study of clad steel welds is quite complex, since usually several passes of arc welding are carried out with different fillers for base steel and the cladding layer, as shown in the previous section. Furthermore, in fusion welding processes, such as weld overlay or welding between two clad steel plates, dilution plays a fundamental role in determining the molten pool composition, on which depend the joint properties.

The following relationships give the dilution ratios in the weld, with respect to the filler metal (d_F_) and one or possibly two metals (d_M1_ and d_M2_) that take part in the fusion process:

d_F_ = A_F_/(A_F_ + A_M1_ + A_M2_), (1)

d_Mi_ = A_Mi_/(A_F_ + A_M1_ + A_M2_), (2)

where A_F_, A_M1_ and A_M2_ represent the volumetric contributions to the weld of the filler and the two base metals; d_Mi_ and A_Mi_ represent the dilution ratio and the volumetric contribution to the weld of one of the base metals, respectively. These quantities can be reduced by one dimension into area terms under the assumption that the cross-sectional areas do not vary along the weld bead length [71].

The dilution rate varies for each welding process. However, since for the same welding process the dilution rate can vary depending on working parameters (such as power, travel speed, preheating or inter-pass temperature), it is necessary to take each specific case into consideration. By way of example, Table 2 collects, for some processes of weld overlaying, the dilution values expressed as percentages. The wide span of the reported quantities is due to variability in the process parameters. In the same table, slightly more accurate ranges of dilution values are reported in the case of cladding with a high Ni alloy, expressed as Fe (%) diffused from the base steel, specifying the corresponding thicknesses of the cladding layers obtained with a single pass.

As for the LBW overlay process proposed in [53], austenitic steel plates were remelted onto the carbon steel substrate, which contribute, by melting in turn, to the composition of the final layer. This process was compared to cold metal transfer (CMT), based on adding a traditional filler wire through GMAW using a pulsing action instead of continuous power. In this way, the heat input in CMT is lower than that in conventional GMAW, offering a significant benefit due to the reduced thermal effects [73]. 

The two processes carried out in [53] (LBW and CMT) showed the same dilution: a low value for the base metal (d_M_ less than 20%) and a greater one for the filler (d_F_ greater than 80%). When these values are known, the layer final composition can be calculated by weighted mass balance. 

**Figure 14:**
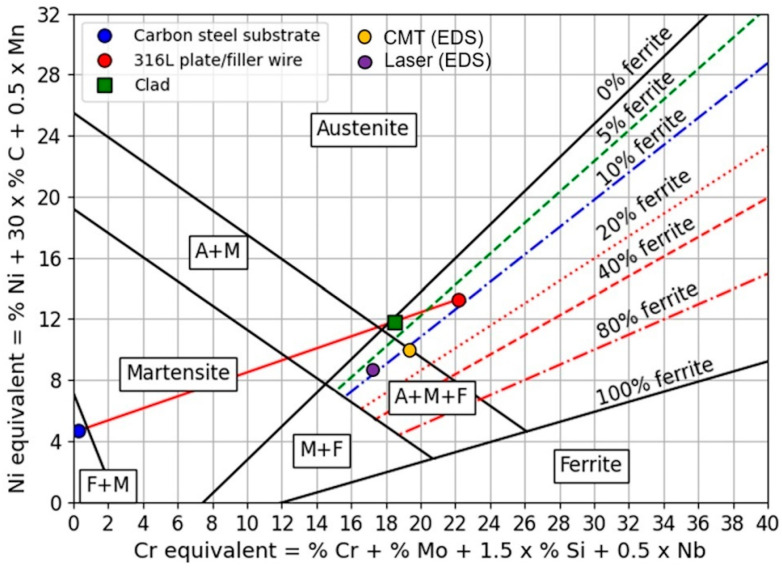
Schaeffler diagram with the representative points of the carbon steel substrate, AISI 316 filler (plate or wire) and clad layer (resulting from a mass balance for a carbon steel dilution d_M_ = 17%). The experimental compositions of the clad layer, obtained with SEM-EDS measurements, for CMT and the laser beam process are given too. Reproduced from [53].

The resulting microstructure can be estimated through the Schaeffler diagram in Figure 14, based on equivalent compositions expressed as percentages by weight. The representative point of the layer obtained by CMT or the laser beam process indicates that the microstructure tends to be almost fully austenitic with 2–3% of residual δ-ferrite for both the processes, whereas SEM-EDS measurements showed that the two processes have distinct representative points with a ferrite content equal to about 10%, a value close to that estimated from the metallographic observations [53]. This discrepancy can be ascribed to the effect of the solidification rate, which is not considered in the Schaeffler diagram. 

Dilution is responsible for fusion zone composition and consequently for the weld metallurgical and mechanical features. This topic has been widely addressed in the literature, and many studies have been specifically aimed at investigating the solidification modes of the weld metal (see, for reference, [74]).

In general, the composition range of austenitic steels is broad enough that four types of solidification modes and subsequent solid-state transformations can occur, as reassumed in Table 3 in agreement with what has been reported by several authors [75,76]. To facilitate reading, the composition limits for each solidification mode have been expressed as a function of the Cr_eq_/Ni_eq_ ratio, in which Cr_eq_ and Ni_eq_ can have the same expressions as those utilised for the Schaeffler diagram [77]. Moreover, Table 3 shows the final microstructures resulting from each solidification mode. 

It is important to note that the A and AF solidification modes are associated with primary austenite solidification, while the FA and F types have δ-ferrite as the primary phase. The presence of this phase in the final stage of the FA solidification mode will be discussed in the following, highlighting how it provides the best resistance to solidification cracking.

For the composition range in which solidification occurs following the FA mode, the Welding Research Council (WRC) diagram (version revised in 1992) provides detailed results (according to Cr_eq_ = Cr + Mo + 0.7∙Nb and Ni_eq_ = Ni + 35∙C + 20∙N + 0.25∙Cu) for determining the ferrite percentage of the final microstructure and is therefore currently utilised in the literature [78,79,80].

**Figure 15:**
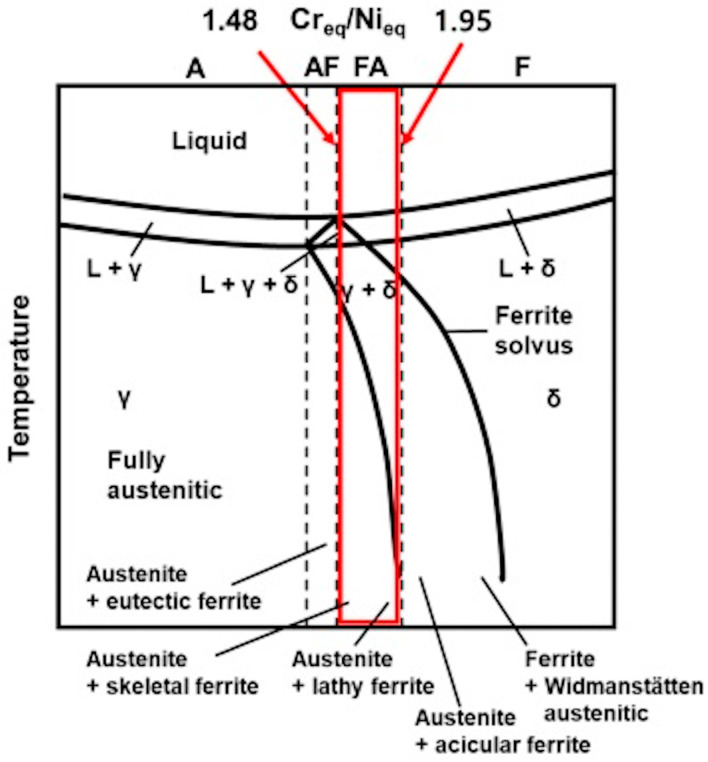
Fe–Cr-Ni pseudo-binary diagram (70% of iron) with indication of the FA mode composition range. Reproduced from [82].

To highlight the different microstructures due to the physical transformations that occur during solidification and subsequent cooling, several authors [81,82,83] considered in their studies the Fe-Cr-Ni pseudo-binary diagram with 70% iron by weight, which can be represented according to the Cr_eq_/Ni_eq_ ratio, as shown in Figure 15 where the limits for the FA mode are indicated by the red rectangle.

**Figure 16:**
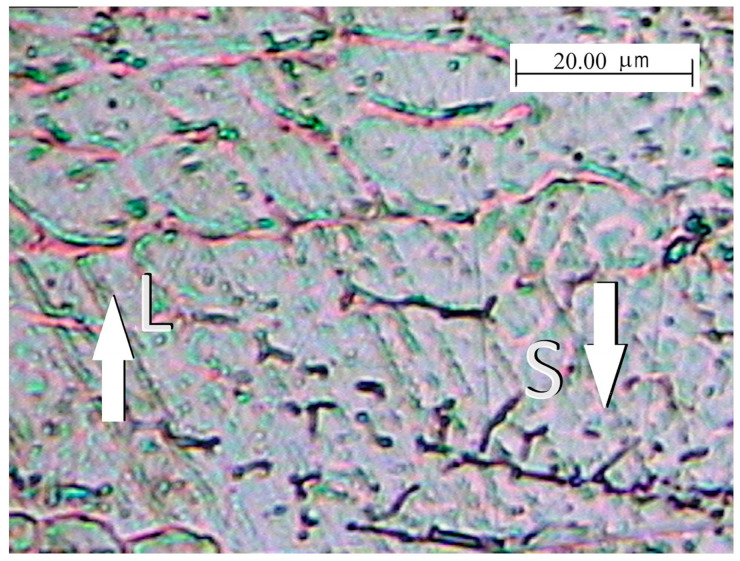
Detail of a fused zone (Cr_eq_/Ni_eq_ = 1.63) with indication by the white arrows of the skeletal and lathy residual ferrite. Reproduced from [94].

The different expressions of Cr_eq_ and Ni_eq_, adopted for the Schaeffler and WRC 1992 diagrams, produce small deviations in the prediction of the phases, as shown in the work by Kianersi et al. [84]. In any case, the predictions made by the aforementioned diagram are not exhaustive, since the δ → γ solid-state transformation is a diffusion-controlled process depending on the cooling rate [85]. As a matter of fact, when the welding heat input increases, the cooling rate is reduced [86]; consequently, the duration of the δ → γ transformation is prolonged, leading to the reduction in the final amount of residual δ-ferrite. Conversely, a lower heat input leads to faster cooling, reduced time for the δ → γ transformation and therefore a greater amount of residual δ-ferrite [87]. In this regard, numerical and analytical models, as well as experimental methods, were recently developed to simulate the effects of welding parameters on thermal fields and cooling rates [88,89,90], as well as on residual stress [91,92], although there is a lack of articles in the literature that deal specifically with welding of clad steel plates. In any case, the cooling rate is a critical parameter as it affects the morphology of ferrite: skeletal ferrite is favoured by a low value (high heat input) while lathy columnar ferrite is favoured by a higher value (lower heat input) [93]. In particular, these morphologies can coexist in the same weld (Figure 16), with one or the other prevailing depending on the cooling rate [94].

The formation of weld solidification cracking depends on the solidification mode and therefore is a function of composition: it has been ascertained that the FA mode offers the greatest resistance to cracking in the finale stage of solidification, whereas the F mode is more susceptible to this phenomenon, but its behaviour is better than those of the A and AF ones [74]. In this regard, it is should be noted that, for austenitic compositions, the weld susceptibility to solidification cracking is affected by the grain boundary morphology, since primary austenite solidification (AF mode) generates straight boundaries that are easy to follow by cracks during their growth; conversely, the tortuous ferritic boundaries that characterise the FA mode produce some beneficial effects, making the growth of solidification cracks difficult [95] and also preventing hydrogen embrittlement [87]. 

In any case, the concentration limit of 10% for residual ferrite should not be exceeded because its excessive presence significantly reduces toughness (especially at cryogenic temperatures), deteriorates corrosion resistance and favours the formation of brittle intermetallic phases at high service temperatures [78]. Indeed, when working temperatures are in the 550–900 °C range, primary ferrite can decompose into carbides and phases detrimental for mechanical properties, such as ductility, impact toughness and creep strength as well as corrosion resistance [96]. 

Hybrid arc welding processes in multiple passes with the use of different filler metals could give rise to dissimilar microstructures; in general, the microstructure of the layers close to the base metal is ferritic/pearlitic, while close to the cladding steel, where fillers rich in Ni are used, it is austenitic with residual ferrite (see, for example, Figure 4 of the article by Ghorbel et al. [58]).

The presence of martensite, considered a welding defect since it tends to trigger cracks under fatigue, can be observed in the ferritic layers deposited on the austenitic ones. It forms due to dilution, which enhances the presence of alloy elements, such as Cr and Ni, and consequently causes the shift of the CCC curve and the decrease in the critical martensitic cooling rate [60]. Similar cases of martensite formation were reported in [61,62]. In [63], the formation of martensite is ascribed to values of dilution greater than 60%, which lead to the FZ in the A + M field of the Schaeffler diagram (Figure 14).

When welding using a filler metal with a composition dissimilar to that of the base metal, the diluted composition of the weld can be assumed homogeneous, due to strong hydrodynamic mixing forces in the molten pool. However, along the fusion line, the formation of very thin PDZ with an intermediate composition between the base metal and the bulk weld metal is usually observed [97]. 

**Figure 17:**
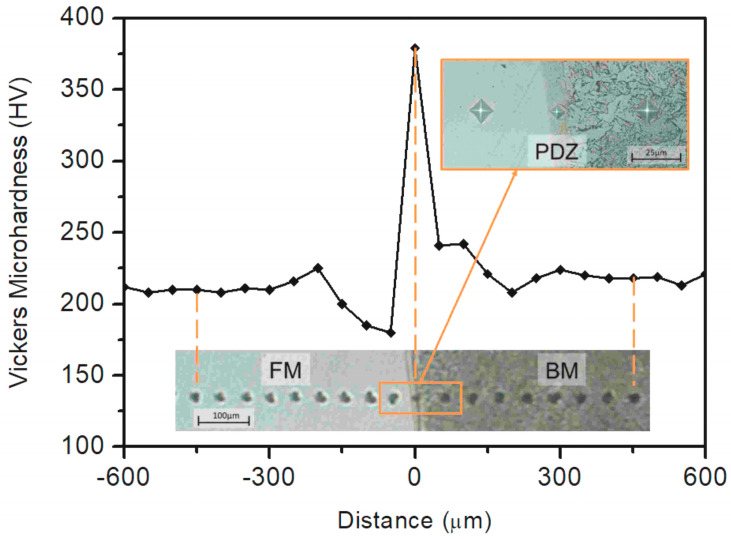
Vickers microhardness profile along a transversal line across the interface, from BM (on the left) to FM (on the right). Reproduced from [99].

As documented by some authors [98,99], a carbon-enriched PDZ, characterised by a hard martensitic microstructure, occurs when carbon steel base material is weld overlaid by high Ni alloy filler metal. In Figure 17, the AST A36 carbon steel base metal (BM), the AWS ER NiCrMo-3 (Inconel 625) filler metal (FM) deposited by GMAW and the interposed PDZ are outlined by the results of the Vickers microhardness test; in this case, the narrow peak around 380 HV testifies to the presence of a martensitic microstructure in the PDZ.

Another issue to be considered concerns the presence of decarburization and carbon accumulation zones at the melting interface between the layers deposited with different compositions at the level of the carbon steel/austenitic steel transition zone, while martensite is formed in the substrate by diffusion of the alloying elements, more markedly as the welding heat input increases [64].

**Figure 18:**
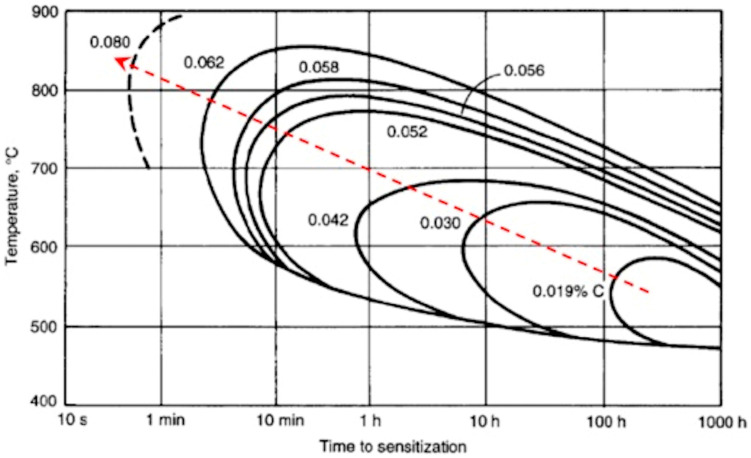
Precipitation curves of Cr_23_C_6_ carbide as a function of carbon content. The dashed red line indicates how the precipitation kinetics become faster as the carbon content increases. Reproduced from [101].

In addition to the effect on martensitic transformation, carbon accumulation generates conditions favourable to Cr-carbide precipitation. It results in a local hardness increase, which weakens the microstructure, as well as in sensitization to intergranular corrosion (see [100] for a review). In this regard, Figure 18 shows the temperature–time sensitization curves [101], and the red dashed line indicates the effect of an increase in carbon percentage; it can be deduced that the precipitation time is significantly reduced when the carbon content increases from 0.03%, which is the typical value of the AISI 304L composition, to higher values, making the precipitation of carbides possible.

It can be deduced that the occurrence of sensitization is possible in adjacent layers where dilution may result in an increase in carbon content, Therefore, to avoid a long permanence of the weld within the critical range of temperature, a cooling time should be allowed after each pass, and the subsequent pass should initiate only when the highest temperature of the weld reaches 150 °C [59].

At the austenitic layer side, away from the cladding line, the HAZ does not undergo conditions favourable for carbide precipitation, requiring, e.g., for C = 0.03%, a permanence of approximately 8 h at 600 °C or 30 h at 500 °C (Figure 18). Such long time intervals can be excluded by means of the simulation carried out in [90] on the thermal field arising during LBW of thick AISI 304 plates.

**Figure 19:**
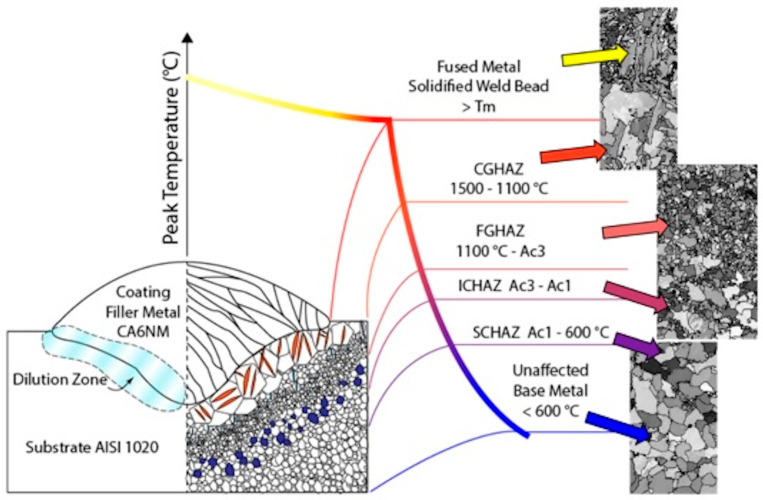
Schematic representation of welded microstructures and their metallurgical zones, divided according to the peak temperature. Images of the generated microstructure are displayed accordingly. Reproduced from [45].

At the base material side, carbon steel shows the characteristic transformations that occur in the HAZ. By way of example, in Figure 19 four regions are highlighted based on the temperatures reached [45], namely coarse-grained (CGHAZ), fine-grained (FGHAZ), inter-critical (ICHAZ) and subcritical heat-affected zones (SCHAZ), as well as the unaffected zone. They can present martensitic and bainitic phases where sufficiently high cooling rates are reached during the welding process.

## 5. Conclusions and Future Directions

This article reviewed the scientific literature on the production processes of clad steels and in particular on fusion welding of ferritic/austenitic plates. The welding processes based on multiple passes of traditional electric arc procedures are currently carried out in the petrochemical and energy industry. Alongside them, the most modern laser beam or hybrid arc–laser welding processes are increasingly used, as they allow the joining of thick plates in a single pass, which would not be possible with the usual techniques.

Over the last few decades, researchers’ interest has been focused on the typical metallurgical issues that characterise the behaviour of ferritic and stainless steels. Progress in this field has developed over the last 20 years, as highlighted by the recent scientific literature. The majority of the articles examined (76/101) date back to the last five years, demonstrating the current interest in this topic.

Some issues of the production processes concern diffusion at the clad steel/base steel interface in hot rolling, formation of intermetallic compounds at the Al/steel interface in explosive cladding and dilution at the deposited cladding layer/base steel interface in weld overlay.

As for fusion welding, some issues requiring attention from researchers have emerged in this review:

- Dilution between the filler and base metal at the level of the base and clad steel, as well as dilution between the deposited layers in multi-pass welding;
- Unexpected composition of the welds;
- Solidification in non-optimal mode to ensure crack-free welds;
- Carbide enrichment in the ferritic phase, followed by martensitic transformation during cooling;
- Carbide precipitation in the austenitic phase, followed by local hardening and sensitization to intergranular corrosion as a function of the time permanence within the critical interval of temperature.

High-penetration LBW allows joining of clad steel in a single pass, even if some attention is required in the setup of the filler material to prevent dilution between the base metal and the cladding metal from causing unwanted effects.

Future work should be aimed at verifying the possibility of industrially adopting solutions capable of overcoming these shortcomings, such as the use of consumable inserts for single-pass laser welding. Moreover, further investigations are still needed on the choice of arc welding systems and filler metals suitable for clad and base steel, respectively, as well as on the edge preparation, the sequence of the multiple passes, the thermal cycle effects on the weld metallurgy and the possible occurrence of distortions or residual stresses. In this regard, it is also desirable that simulation methods are developed for the prediction of thermal fields capable of taking into account the complexity of the phenomena involved in clad steel welding.

## Figures and Tables

**Table 1:** Characteristics of the welding processes.

Welding Process	Welding Procedure	Welding Sequence	Filler Geometry	Weld Cross-Section Shape	Dilution Rate
Arc welding	Multi-pass	Starting from base metal	Wire	Wide	High
LBW	Single pass	-	Wire/strip	Narrow	Low
Hybrid (laser/arc)	Single pass	-	Wire/strip	Very wide at arc side	Low at laser side/high at arc side

**Table 2:** Dilution, Fe (%) diffused from base steel and cladding layer thickness for some weld overlay processes.

Weld Overlay Process	Dilution (%) [40]	Fe (%)/Cladding Layer Thickness (mm) [72]
SAW	30–70	-
GMAW	13–33	13–26.9/3–3.5
SMAW	13–20	22–23.4/3–4
GTAW	13–20	12.6/-
GTAW-HW	10–40	6.6–23.7/1.6–3.5
CMT	-	1–4.1/3

**Table 3:** Solidification modes, phase transformations, composition limits and corresponding microstructures.

Solidification Modes	Transformations	Composition Limits (Cr_eq_/Ni_eq_)	Microstructures
Austenitic (A mode)	L → L + γ → γ	<1.25	Fully austenitic
Austenitic/ferritic (AF mode)	L → L + γ → L + δ + γ → γ + δ	1.25–1.48	Austenitic with ferrite at cell and dendrite boundaries
Ferritic/austenitic (FA mode)	L → L + δ → L + δ + γ → δ + γ	1.48–1.95	Skeletal and/or lathy ferrite from F-A solid-state transformation
Ferritic (F mode)	L → L + δ → δ + γ ^1^	>1.95	Ferrite matrix and Widmastänstatten austenite

^1^ A high Cr content leads to a full ferritic final microstructure. As temperature decreases, ferrite may transform into the brittle σ phase.

## Data Availability

No new data were created; therefore, data sharing is not applicable to this article.
